# Transcranial Doppler Ultrasonography in Beta-thalassemia Major Patients Without and With Thrombocytosis

**DOI:** 10.5812/ircmj.4606

**Published:** 2013-03-05

**Authors:** Abdolhamid Shariat, Masoume Nazeri, Amin Abolhasani Foroughi, Mehran Karimi

**Affiliations:** 1 Department of Neurology, Nemazee Hospital, Shiraz University of Medical Sciences, Shiraz, IR Iran; 2 Medical Imaging Research Center, Department of Radiology, Nemazee Hospital, Shiraz University of Medical Sciences, Shiraz, IR Iran; 3 Hematology Research Center, Shiraz University of Medical Sciences, Shiraz, IR Iran

**Keywords:** Beta-thalassemia, Thrombocytosis, Ultrasonography, Doppler, Transcranial

## Abstract

**Background:**

Beta-thalassemia is a type of anemia in which the patients may require splenectomy and this can lead to thrombocytosis with increased risk of stroke. Transcranial Doppler ultrasound is a method for determining cerebral vessel stenosis.

**Objectives:**

The aim of this study was to investigate whether the risk of a future stroke secondary to cerebral artery stenosis can be predicted with the use of transcranial Doppler ultrasound in beta-thalassemia major patients.

**Patients and Methods:**

This study included 54 beta-thalassemia major patients divided into 2 groups; group A consisted of 28 patients who have thrombocytosis secondary to a previous splenectomy and group B comprised of 26 patients who did not have a splenectomy with normal platelet count, as well as a control group of 30 healthy individuals.

**Results:**

Transcranial Doppler ultrasound of the cerebral vessels were performed in all participants, and the results for each group were compared with the controls. In addition, patients were evaluated for evidence of high flow velocity in the cerebral vessels that met the clinically significant criteria of ≥ 50% stenosis. Transcranial Doppler ultrasound velocity criteria for > 50% stenosis, indicating a risk of stroke, were not documented in any patients but increase in cerebral blood velocities in many arteries in group A and in some arteries in group B were revealed.

**Conclusion:**

Following splenectomy, thrombocytosis can predispose the patients to an increase in cerebral blood velocities more than respected with anemia. But by transcranial doppler ultrasonography no evidence of significant stenosis were found in intracerebral arteries to conclude that the beta-thalassemia major patients were more prone to the development of stroke secondary to this abnormality.

## 1. Background

Thalassemia is a congenital disease caused by a partial or complete error in the synthesis of alpha or beta chain of hemoglobin, resulting in anemia. If the error is inherited in a homozygous pattern, anemia is severe and the patient experiences severe disease from the early years of life. Treatment is based on long-term blood transfusion, which increases the patient’s life expectancy. One undesirable side effect of chronic transfusion is iron overload, but recent improvements in medical therapy for this condition have mitigated this problem. Other complications have now acquired more importance. One of these is a hypercoagulable state, particularly in patients with thalassemia intermedia (1, 2). Cerebrovascular accidents have been extensively reported in thalassemia patients (3).The hypercoagulable state occurring in many thalassemia patients is caused by thrombocytosis, defects in coagulation inhibitors, cardiac and liver dysfunction, hypothyroidism or thrombocytosis secondary to splenectomy (4-6). Hypercoagulation is more prevalent in patients who have not received regular transfusion, (2) although transfused patients more commonly experience hypertension, seizures and cerebral hemorrhage(2). Other hypercoagulation complications often seen in beta-thalassemia major patients include deep venous thrombosis, pulmonary emboli and recurrent arterial occlusion. In patients who have undergone splenectomy, there is a greater prevalence of venous thrombosis (2). Sickle cell anemia is a hemolytic anemia caused by a mutation in an amino acid of the beta chain (4). One of the complications of sickle cell disease is stroke, which occurs in 7% to 8% of children with this condition (7-9). Based on the flow velocity criteria for stenosis, the risk of stroke can be assessed by transcranial Doppler ultrasound (TCD) (10-12). This technique can be used to determine the need for prophylactic drug therapy in these patients as a preventive measure (13). Prompted by the beneficial use of TCD in sickle cell disease, our aim in this study was to apply TCD for the determination of stroke risk in beta-thalassemia major (TM) patients. To our knowledge, TCD has not been used previously for this purpose. We hypothesized that TCD could be of value in TM patients who require splenectomy, considering the increased risk of thromboembolic events reported in this population (14). We guess by using TCD to determine stroke risk in TM patients, the role of blood transfusion and the effect of splenectomy in increasing the chance of stroke can be examined. Furthermore, stroke risk can be considered in relation to the indications for splenectomy in these patients.

## 2. Objectives

The aim of this study was to investigate whether the risk of a future stroke secondary to cerebral artery stenosis can be predicted with the use of transcranial Doppler ultrasound in beta-thalassemia major patients.

## 3. Patients and Methods

This study was conducted in our institute, over a 1-year period, from October 2009 to October 2010. Sixty TM patients seen in our Thalassemia Center for routine follow-ups were studied. The inclusion criteria were as follows: patients of both sexes were at least 18 years old with transfusion-dependent TM, no history of thrombosis or cardiovascular accident and a normal neurological examination. The diagnosis of TM was based on a complete blood count and hemoglobin electrophoresis. All patients had been receiving regular blood transfusions at 2 to 4week intervals, starting when they were younger than 2 years old. Patients were divided into 2 groups according to their splenectomy status and platelet count: Group A included TM patients who had undergone splenectomy and had a platelet count of more than 500,000/mm3, and Group B was comprised of TM patients who had not undergone splenectomy and did not have thrombocytosis. The control group comprised of thirty healthy sex-matched adults. Four patients who had thrombocytosis, but had not undergone splenectomy and 2 patients who had undergone splenectomy, but did not have thrombocytosis were excluded from the study. Thus, 28 patients were included in group A and 26 patients in group B. None of the studied patients had hypertension, diabetes mellitus, ischemic heart disease, previous ischemic stroke or any history of documented thrombosis. Patients gave written informed consent to participate and the study was approved by the Medical Ethics Committee of our University of Medical Sciences. Blood sampling and laboratory analyses were performed according to the hospital follow-up protocol for these patients. The complete blood count was recorded. The hematologist referred the patients to a neurologist for TCD. The neurologist measured Time Average Mean Velocity (TAMV) in the middle cerebral artery (MCA), anterior cerebral artery (ACA), internal carotid artery (ICA), posterior cerebral artery (PCA), and vertebrobasilar system. In sickle cell patients, TAMVs are increased because of anemia and vasodilatation caused by cerebral tissue hypoxia. For this reason, we did not use the velocity criteria established in the STOP study to determine vessel stenosis in our beta-thalassemia patients. Instead, we applied the velocity criteria shown in Table 1 (2, 13, 15) to define an abnormal transcranial Doppler result in our population. If an increase in mean velocities in any of the arteries were noted, comparison to the same segment of the vessel -on the other side and velocity ratio to proximal segment of the same artery more than doubling time were considered. All patients were examined by a single neurologist with experience in performing transcranial Doppler ultrasonography, always using the same equipment (Legend TC22/E, 2 MHz probe). Any abnormal results were reproduced by repeating the TCD examination on another day. Data were analyzed by SPSS (V.15), using the Student t test for comparison of age and hemoglobin level between the two groups. Chi-square test was done to compare sex between case and control groups. ANCOVA was used to compare mean flow velocity of each of the evaluated arteries among group A, group B and control group. P < 0.05 was considered statistically significant.

**Table 1.** Velocity Criteriafor Defining Abnormal Transcranial Doppler Ultrasound

Artery	Abnormal Mean Flow Velocity, cm/s	Time Average Mean Velocity Criteria for ≥ 50% Stenosis
**MCA^a^**	≥ 80 cm/s	≥ 100 cm/s or prestenotic to stenotic velocity ratio ≥ 2
**ACA^a^**	≥ 80 cm/s	Not applicable
**ICA^a^**	≥ 70 cm/s	≥ 90 cm/s or prestenotic to stenotic velocity ratio ≥ 2
**PCA^a^**	≥ 50	Not applicable
**BA^a^**	≥ 60 cm/s	≥ 80 cm/s or prestenotic to stenotic velocity ratio ≥ 2
**VA^a^**	≥ 50 cm/s	≥ 80 cm/s or prestenotic to stenotic velocity ratio ≥ 2

^a^Abbreviations: ACA: Anterior Cerebral Artery, BA Basilar Artery, ICA: Internal Cerebral Artery, MCA: Middle Cerebral Artery, PCA Posterior Cerebral Artery, VA Vertebral Artery

## 4. Results

Sixty patients (55% women) with a mean age of 23 years (range 18-32) were enrolled in the study. Thirty healthy sex matched adults (53% women) (P = 1.0) with a mean age of 25 years were included as controls. The mean age of the control group was about 2 years higher than that of the patients (P = 0.003). Twenty-eight patients who had undergone splenectomy and had thrombocytosis (platelet count > 500,000) were included in group A. Twenty-six patients who had not undergone splenectomy and had normal platelet counts were included in group B. The mean, maximum and minimum of TAMVs recorded on TCD for each artery in the two patient groups and the comparisons with the controls are shown in Tables 2, 3, 4 and 5. The right and left vertebral artery TAMVs in group B were significantly higher than the values in the control group ( P < 0.001in both), but there were no significant increases in TAMVs of the right or left ICA, MCA, ACA, PCA and BA in group B with respect to the controls. In group A, TAMV in the left PCA (P = 0.229) and basilar artery (P = 0.601) showed no significant differences as compared to the control group (P > 0.05), but flow velocities in both ACAs, MCAs, ICAs and vertebral arteries as well as in the right PCA were significantly higher in group A than in the controls. In 8 patients in group A, flow velocities were above 90 cm/s in the left or right ICA and above 100 cm/s in the left or right MCA. However, no spectral broadening and no velocity ratio greater than two times with respect to proximal segment and no increase in mean velocity more than 30% comparing to the other side were noted; thereby, none were meeting the criteria for ≥ 50% stenosis. In 2 patients from group B, ICA and MCA flow velocities were more than 90cm/s and 100 cm/s respectively, but again they did not fulfill the other criteria for vessel stenosis. Average hemoglobin level was 9.38 ± 1.08 in group A and 9.43 ± 0.81 in the group B, with no significant differences (P = 0.85).

**Table 2.** Middle Cerebral Artery (MCA) mean flow velocity results in groups A and B and comparison with the controls

Vessel	TAMV ^a^, cm/s, Mean ± SD	Maximum value of TAMV, cm/s	Minimum value of TAMV, cm/s	P value
**Right MCA**				
Group A^b^	69 ± 19	105	36	< 0.001
Group B^c^	56 ± 16	100	35	0.232
Control	49 ± 8	63	37	
**Left MCA**				
Group A^b^	70 ± 20	102	41	< 0.001
Group B^c^	49 ± 15	94	27	0.407
Control	57 ± 9	79	37	

^a^ Time Average Mean Velocity

^b^ Splenectomy + Thrombocytosis

^c^ No splenectomy + Normal platelet count

**Table 3.** Anterior Cerebral Artery (ACA) flow velocity results in groups A and B and comparison with the controls

Vessel	TAMV ^a^,cm/s, Mean ± SD	Maximum value of TAMV, cm/s	Minimum value of TAMV , cm/s	P value
**Right ACA**				
Group A^b^	52 ± 13	82	32	0.013
Group B^c^	47 ± 10	66	27	0.726
Control	42 ± 8	62	29	
**Left ACA**				
Group A^b^	53 ± 14	85	29	0.027
Group B^c^	47 ± 11	76	25	0.790
Control	44 ± 9	68	25	

^a^Time Average Mean Velocity

^b^Splenectomy + Thrombocytosis

^c^No splenectomy + Normal platelet count

**Table 4.** Internal Cerebral Artery (ICA) flow velocity results in groups A and B and comparison with the controls

Vessel	TAMV ^a^, cm/s, Mean ± SD	Maximum value of TAMV, cm/s	Minimum value of TAMV, cm/s	P value
**Right ICA**				
Group A^b^	66 ± 28	117	25	0.002
Group B^c^	51 ± 16	89	29	0.968
Control	50 ± 8	64	26	
**Left ICA**				
Group A^b^	67 ± 27	116	24	0.003
Group B^c^	51 ± 18	92	25	0.899
Control	52 ± 9	66	32	

^a^Time Average Mean Velocity

^b^Splenectomy + Thrombocytosis

^c^No splenectomy + Normal platelet count

**Table 5.** PosteriorCerebral Artery (PCA) and Vertebral Artery (VA) and Basilar Artery (BA) flow velocity results in groups A and B and comparison with the controls

Vessel	TAMV ^a^, cm/s, Mean ± SD	Maximum value of TAMV, cm/s	Minimum value of TAMV, cm/s	P value
**Right PCA**				
Group A^b^	32 ± 7	45	19	0.021
Group B^c^	28 ± 7	43	13	0.822
Control	27 ± 6	40	18	
**Left PCA**				
Group A^b^	32 ± 8	51	21	0.229
Group B^c^	28 ± 6	39	17	0.281
Control	29 ± 7	44	17	
**Right VA**				
Group A^b^	42 ± 7	58	26	< 0.001
Group B^c^	44 ± 11	79	28	< 0.001
Control	29 ± 5	38	22	
**Left VA**				
Group A^b^	43 ± 8	67	32	< 0.001
Group B^c^	42 ± 9	70	28	< 0.001
Control	29 ± 5	37	22	
**BA**				
Group A^c^	52 ± 12	72	28	0.601
Group B^c^	54 ± 12	81	30	0.756
Control	47 ± 8	58	20	

^a^Time Average Mean Velocity

^b^Splenectomy + Thrombocytosis

^c^No splenectomy + Normal platelet count

## 5. Discussion

In this study we grouped the cases according to the occurrence of thrombocytosis secondary to splenectomy to investigate the effect of an increase in platelet count on predisposition to cerebrovascular accidents. TCD velocity criteria for > 50% stenosis were not documented in any cerebral vessel in the 54 TM patients studied. Cerebral vessel mean flow velocity measurements in TM patients who did not undergo splenectomy showed only mild increases in the right and left vertebral arteries that did not meet the criteria for ≥ 50% stenosis. In contrast, TM patients with thrombocytosis as a result of previous splenectomy showed higher velocities in all cerebral vessels except for the left PCA and BA. Anemia is a known factor for increase in cerebral mean blood velocities in most of the intracerebral arteries, because a decrease in hemoglobin concentration should be mitigated through an increase in delivery of red blood cells by increasing blood velocity (9-12). Although increase in TAMVs in many cerebral arteries in group A could be due to anemia, yet increase in TAMWs is seen in more arteries with respect to group B. Considering that none of the patients in both groups had vessel stenosis, and the only difference between the two groups were thrombocytosis, we hypothesize that, “thrombocytosis may increase cerebral blood velocities in TM patients after splenectomy”. For documentation of this possibility, planning more studies is required which would follow the patients for several years to look for any evidence of persistence of an increase in cerebral blood velocities or development of possible future stenosis. Furthermore, documentation of vessel abnormality with other imaging modalities such as computed tomography angiography (CTA) or magnetic resonance angiography (MRA) can be helpful. It is well known that regular blood transfusion is a beneficial method for lowering down the chances of stroke in sickle cell anemic patients (13, 16) while in this study we did not find risks of stroke in our TM patients. One explanation could be that the patients were on regular blood transfusion since their early childhood. We are not certain why transfusion can mitigate the risk of stroke in these anemic patients, however increase in hemoglobin levels and lowering of the abnormal beta hemoglobin chains in red blood cells as well as refreshing the interactions between healthy red blood cells and the vessel wall could be possible explanations (16). It must be mentioned that by only using TCD no one can confidently predict stroke risk in patients. However, TCD informs us about the health of more proximal larger intracerebral arteries, but evaluation of the distal segments of cerebral arteries and the cortical arteries are not possible. For better assessment of vessel abnormality, larger studies and assessment of microembolic signals and correlation of TCD results by other imaging modalities such as CTA and MRA could be helpful.

## References

[1] Aessopos A, Kati M, Meletis J (2007). Thalassemia intermedia today: should patients regularly receive transfusions?. Transfusion..

[2] Eldor A, Rachmilewitz EA (2002). The hypercoagulable state in thalassemia.. Blood..

[3] Borgna Pignatti C, Carnelli V, Caruso V, Dore F, De Mattia D, Di Palma A (1998). Thromboembolic events in beta thalassemia major: an Italian multicenter study.. Acta Haematol..

[4] Ataga KI, Cappellini MD, Rachmilewitz EA (2007). Beta-thalassaemia and sickle cell anaemia as paradigms of hypercoagulability.. Br J Haematol..

[5] Karimi M, Khanlari M, Rachmilewitz EA (2008). Cerebrovascular accident in beta-thalassemia major (beta-TM) and beta-thalassemia intermedia (beta-TI).. Am J Hematol..

[6] Panigrahi I, Agarwal S (2007). Thromboembolic complications in beta-thalassemia: Beyond the horizon.. Thromb Res..

[7] Ataga KI, Cappellini MD, Rachmilewitz EA (2007). Beta-thalassaemia and sickle cell anaemia as paradigms of hypercoagulability.. Br J Haematol..

[8] Adams RJ (1995). Sickle cell disease and stroke.. J Child Neurol..

[9] Bulas DI, Jones A, Seibert JJ, Driscoll C, O'Donnell R, Adams RJ (2000). Transcranial Doppler (TCD) screening for stroke prevention in sickle cell anemia: pitfalls in technique variation.. Pediatr Radiol..

[10] Deane CR, Goss D, O'Driscoll S, Mellor S, Pohl KR, Dick MC (2008). Transcranial Doppler scanning and the assessment of stroke risk in children with HbSC [corrected] disease.. Arch Dis Child..

[11] Bulas D (2005). Screening children for sickle cell vasculopathy: guidelines for transcranial Doppler evaluation.. Pediatr Radiol..

[12] Lowe LH, Bulas DI (2005). Transcranial Doppler imaging in children: sickle cell screening and beyond.. Pediatr Radiol..

[13] Neish AS, Blews DE, Simms CA, Merritt RK, Spinks AJ (2002). Screening for stroke in sickle cell anemia: comparison of transcranial Doppler imaging and nonimaging US techniques.. Radiology..

[14] Lee MT, Piomelli S, Granger S, Miller ST, Harkness S, Brambilla DJ (2006). Stroke Prevention Trial in Sickle Cell Anemia (STOP): extended follow-up and final results.. Blood..

[15] Taher A, Isma'eel H, Mehio G, Bignamini D, Kattamis A, Rachmilewitz EA (2006). Prevalence of thromboembolic events among 8,860 patients with thalassaemia major and intermedia in the Mediterranean area and Iran.. Thromb Haemost..

[16] Annbelle L, Vijai KS, Miral K, Andrei VA, Mcgahan JP, Goldberg BB (2008). Diagnostic criteria for transcranial doppler ultrasound.. Diagnostic Ultrasound..

[17] Adams RJ, McKie VC, Hsu L, Files B, Vichinsky E, Pegelow C (1998). Prevention of a first stroke by transfusions in children with sickle cell anemia and abnormal results on transcranial Doppler ultrasonography.. N Engl J Med..

